# The Influence of Social Media and Institutional Trust on Vaccine Hesitancy in France: Examining Direct and Mediating Processes

**DOI:** 10.3390/vaccines11081319

**Published:** 2023-08-03

**Authors:** Christopher J. McKinley, Elea Olivier, Jeremy K. Ward

**Affiliations:** 1 School of Communication and Media, Montclair State University, Montclair, NJ 07043, USA; 2 Mathematics Engineering at INSA Rennes, 35700 Rennes, France; 3 Institut National de la Santé et de la Recherche Médicale (INSERM), Université Paris Cité, CNRS, Inserm, Cermes3, F-94800 Villejuif, France

**Keywords:** vaccine hesitancy, social media, institutional trust

## Abstract

Vaccine hesitancy (VH) remains an ongoing challenge in French society. This project explored how institutional trust and preference for information via social media (PISM) drive hesitancy. Across a large, nationally represented population, our findings show that PISM and trust are strongly correlated measures, with both independently predicting VH. Subsequent mediation tests show that social media operates as primarily an indirect contributor to VH through trust. Additional tests involving VH and non-VH typologies revealed that institutional trust consistently predicts greater general support for vaccines and reduced distrust in vaccination. Conversely, PISM directly drives vaccine distrust, with its impact on non-hesitancy fully mediated by institutional trust. Overall, these findings point to the relevance for researchers and public health deciders to address the nature by which people utilize social media information resources and how that interacts with levels of trust for national institutions.

## 1. Introduction

In France, the recent emergence of vaccine-related controversies [1,2] has contributed to the long-term persistence of distrust in institutions. Lack of institutional trust, coupled with increased access to misinformation via non-traditional information sources, can further diminish support for vaccination campaigns [2,3,4]. Although substantial attention has focused on social media as a problematic vaccine information source, recent studies also suggest that concerns over the negative effects of social media misinformation are overstated [5]. The current investigation seeks to explore the interrelationship between institutional trust and social media use drive vaccine hesitancy. Drawing from competing theoretical and empirical arguments, we assess how these factors operate sequentially in broader processes leading to vaccine hesitancy. Across four waves of data collected from nationally representative French samples, this exploratory investigation examines pathways linking institutional trust and preference for information via social media (PISM) to vaccine hesitancy (VH) in France. Within this analysis, we examine distinctions among different subgroups/typologies—vaccine hesitant vs. non-vaccine hesitant individuals—to address the importance social media information preference and institutional trust to vaccine judgments. Overall, this investigation seeks to offer a broader understanding of interrelated role between attitudes towards national institutions and alternative media sources on vaccine decision making. In addition, we address the broader significance of social media as a potential driver of vaccine resistance.

### 1.1. Vaccine Hesitancy in France

Conceptually, vaccine hesitancy (VH) can reflect a broad reluctance towards vaccination in general or reticence targeted at some vaccines in particular [6]. Although scholars have called for clarifying this terminology [7,8], a definition commonly utilized refers to vaccine hesitancy as a “delay in acceptance or refusal of vaccines despite the availability of services” [9] (p. 4163). Prior to the COVID-19 outbreak, France was one of the most vaccine hesitant countries in the world (in terms of negative perceptions) [10,11,12]. Rates of VH have ranged from 25–70% depending on measurement and timing of surveys [13]. Although the current study does not focus on COVID-19 vaccination, it is worth acknowledging that at the onset of COVID-19 vaccine distribution (December 2020), France reported the highest level of COVID-19-VH among European nations, with less than 50% of those surveyed intending to get the COVID-19 vaccine [14]. Although more than 90% of French adults have now received a COVID-19 vaccine [15], large sectors of the French population continue to doubt the safety and efficacy of this vaccine for children and adults [16]. 

Multiple factors contribute to high VH in France, including recommended vaccines being perceived as less important than mandatory ones [17], vaccine-related controversies in France [13], and trust in vaccines and/or institutions [18,19,20]. Furthermore, additional factors linked to VH in other countries—high success rate of previous vaccine campaigns leading to trivilializing virus fears [9] and the spread of misinformation via digital media [21]—may also contribute to French residents being vaccine hesitant. Our investigation centers on the interrelationships between lack of institutional trust—a factor with historical links to VH, and social media—an increasingly utilized information source frequently associated with VH in the literature [6,22]. The current study looks at VH tied to four common vaccines—measles, HPV, hepatitis B, and influenza. Measles and hepatitis B recently (2018) became mandatory in France [1]. HPV is recommended for girls aged 11–21 [23], whereas the seasonal influenza vaccine is recommended for a variety of at-risk populations, including pregnant women, those 65 and older, and obese persons [24].

### 1.2. Institutional Trust

Institutional trust is a reflection of healthy, functioning societies [25,26]. Given that public trust impacts the nature by which residents follow informational resources and utilize services, instilling trustworthiness is critical to both preventing and managing epidemics [27]. Van Bavel, Baicker, and Baggio (2021) argue that strategies needed to contain an epidemic are often difficult to enforce [28]. Consequently, institutional trust becomes increasingly more relevant during large-scale public health crises. Prior research shows that while low levels of government trust contribute to less precautionary behavior during virus outbreaks [29], higher levels of institutional trust predicts increased prevention behavior [30]. 

Recent work has shown that institutional trust directly predicts more negative vaccine attitudes [31]. A systematic review of 25 European countries—including France—showed that trust in health authorities was a central determinant of HPV vaccine hesitancy [32]. The recent history of vaccine-related controversies in France dating back to the late 1990s [13] highlights the struggle to instill public trust. These controversies relate to doubts regarding the safety of adjuvants, the hepatis B vaccine, the HPV vaccine, and the H1N1 vaccine. In particular, during the H1N1 pandemic, the general public pointed to concerns over the marketing of the vaccine and the evaluation of potential side effects [17,33]. Overall, these vaccine-related controversies thrive on a pre-existing lack of institutional trust [34]. Wilson et al. found that French general practitioners (GPs) have themselves expressed distrust in health authorities [34]. This is particularly troubling given the central role GPs play in vaccine implementation/public motivation to vaccinate [35,36].

### 1.3. Social Media 

In contrast to traditional media sources, social media is a relatively inexpensive source to share information. Thought-influencers, who potentially lack topic-specific credentials/expertise, can draw large numbers of followers [37,38], with their perspective being perceived as credible as that of the medical establishment [39]. The sheer size of social media audiences can lead to greater exposure to conspiracy beliefs and other misleading information [3,4]. In support of this argument, research by The Centre for Countering Digital Hate (CCDH) showed that within a one-year period from 2019 through 2020, anti-vaccine social media accounts increased by nearly eight million [40]. In addition, this research showed that those promoting anti-vaccine themes attract substantial followers on both Facebook (roughly 31 million people) and YouTube (17 million). Furthermore, investigations of YouTube and Facebook highlight how vaccine-critical messaging is likely to increase interest/engagement [41,42,43]. Within these platforms, social bots (automated accounts that mimic human beings) operating on social media sites have the capacity to magnify the spread of false information [44,45,46]. The prevalence of these automated accounts—with recent data showing that roughly 9–15% of Twitter accounts are actually social bots [47]—suggests the potential to mislead large numbers of users. Recent analyses assessing French social media environments specifically further support these concerns. Garguilo et al.’s analysis of French-language tweets showed that vaccine critics tend to focus on vaccines perceived to be more controversial [48]. In contrast to pro-vaccine accounts, French vaccine-critical Twitter accounts employ a wider variety of tools to attract interest, including more sources and greater coordination with other hashtags. In an analysis of messaging related to vaccines for pregnant women on social media, Martin et al. found that among sixteen countries, France had the largest percentage of discouraging posts [49]. 

However, while these studies highlight the potential negative impact of social media accounts on the spread of misinformation and potential reinforcement of false beliefs, there are also data indicating less consequential social media effects. For example, Guess et al. found that visits to fake news websites made up a small share of U.S. web users’ Internet diets [5]. These websites were accessed largely by a small subset of U.S. citizens with goals of seeking attitude-confirming content. The researchers argue that effects of fake news exposure may be constrained to increasing support for false claims and consequently that the assumption that fake news has larger effects may be overstated. Several recent studies and commentaries by experts in the field of misinformation studies have also sparked the debate over a possible over-hyping of the effect of fake news on social media [50]. In France, several studies suggest that not only do people have little recourse to social media as a source of information [51], they also tend to be very distrustful of the information circulating on the Internet [52]. Finally, a recent study covering the first year and a half of the COVID-19 epidemic found substantial divergence between the dynamics of the circulation of vaccine-critical discourses on the French Twitter-space and the evolution of intentions to vaccinate against this disease in the French adult population [53].

### 1.4. Exploring Relationships between Institutional Trust and Social Media 

As noted above, social media frequently serves as an outlet for misinformation pertaining to vaccination. Less credible yet widely viewed/shared sources commonly express misleading and/or conspirational views across social media platforms [42,54]. Consequently, although social media content may offer useful insights/information on health-related topics, a greater preference for information exclusively provided via this platform (as opposed to traditional information sources) can be associated with lower levels of institutional trust. Overall, we expect a negative relationship to exist between preference for information via social media (PISM) and institutional trust. In particular, those with greater distrust in institutions are likely to be skeptical of traditional information sources that draw from institutional/mainstream actors. Social media is likely to be an appealing resource as it provides discourses grounded in distrust. 

More consequently, we address how the relationship between PISM and institutional trust drives attitudes towards vaccines. The findings above highlight the implications of diminished institutional trust and increased social media information use on VH sentiment in France. While there exists rationale for bi-directional associations between PISM and trust, prior research offers insights into temporal processes driving VH. One argument, drawn from a social cognitive perspective [55,56], suggests that institutional trust operates as an intervening factor between PISM and VH. Researchers posit that institutional trust derives from previous knowledge of “the trustworthiness of the concerned institutions” [56] (p. 4). Knowledge obtained via media sources—including conspiracies tied to institutions—may precede perceptions of trust [56,57].

Empirical research lends support for these arguments. Findings show that institutional trust emerges from information provided by media sources on government activities [58]. Further analyses show that misinformation drives distrust in health professionals, ultimately leading to delays in seeking preventative care [59,60]. Additionally, greater institutional trust directly predicts favorable vaccine attitudes [56], while mistrust in government sources directly predicts greater VH [61]. Drawing from these findings, PISM may indirectly predict VH via institutional trust. 

A competing argument explores PISM as a direct antecedent to VH. Theoretical arguments from information-seeking and conspiracy literature highlight how individuals may utilize alternative information sources (such as social media) when faced with uncertainty and ambiguity [62,63]. Ultimately, information seeking via social media may offer clarity when individuals perceive that they cannot trust those in positions of authority. Additional theoretical and empirical research identifies lack of institutional trust as an antecedent to increased risk perceptions [64]. By increasing risk perceptions, individuals are increasingly motivated to seek out information. 

Importantly, alternative media channels may be particularly appealing when people distrust institutions. Given French residents’ historical distrust for institutions [20,65,66]—which include traditional media—individuals will turn to alternative information resources (such as social media) for information. Distrust in institutions can drive individuals away from mainstream sources, sources that give too much attention and credit to institutional/mainstream actors. Among the alternative sources of information are the forms of social media where people are very likely to encounter discourses/misinformation grounded in skepticism/distrust. Consequently, information obtained via social media could intervene in the relationship between institutional trust and VH. Recent data have linked anti-vaccine beliefs directly to social media use [67]. Overall, a competing mediation model examines how institutional trust indirectly predicts VH through PISM. 

## 2. Method

### 2.1. Participants 

We conducted four cross-sectional online surveys among representative samples of the French mainland population aged 18 and over, with participants randomly selected from an online research panel of more than 750,000 individuals. Overall, a total of 9177 individuals completed the survey spread across four waves: July 2021 (*n* = 3087), October 2021 (*n* = 2015), December 2021 (*n* = 2022), and May 2022 (*n* = 2053). The researchers employed quota sampling methods to match French census statistics according to age, sex, profession, region of residence, and size of the town. Participants receive points for completing these surveys. Ultimately, participants could exchange points in return for gift cards. Demographic information collected included age, gender, educational level, and income. 

### 2.2. Vaccine Hesitancy

Vaccine hesitancy was calculated through five items measured on a four–point Likert scale (1 = strongly favorable, 4 = strongly unfavorable), with participants also having the option to respond with “I don’t have an opinion”. The vaccine hesitancy measure included one item asking about general favorability towards vaccines and four items addressing favorability towards four specific vaccines: HPV, measles, influenza, and hepatitis B. The five items were summed together, then averaged to create a vaccine hesitancy scale (Cronbach’s alpha = 0.84). The “no opinion” responses were inputted to the mean value of each item. 

### 2.3. Institutional Trust

The researchers assessed institutional trust through five items all beginning with the following stem: “how much trust do you have in”. Participants answered the questions on a 1–4 scale ranging from 1 (no trust at all) to 4 (strong trust). The different institutions included the media, parliament, science, government agencies that control or regulate health and environmental risk, and the government. An institutional trust measure was constructed in similar fashion as the vaccine hesitancy measure (Cronbach’s alpha = 0.81). 

### 2.4. Preference for Information via Social Media 

Finally, we assessed preference for information by asking respondents to indicate their three preferred sources of information out of “television”, “radio”, “print media”, “newspaper websites”, “other websites”, and “online social networks” (Facebook, Twitter, etc.). To develop the “preference for information via social media” (PISM) measure, we created a dichotomous “yes/no” measure based on whether participants had listed online social networks as one of their three preferred sources of information. 

### 2.5. Statistical Analyses

The “R” programming package was employed for all analyses, including bivariate correlations between the central study variables and subsequent multiple regression tests. Mediation tests were performed through the “multilevel” package in R, which combines assumptions from the Sobel test [68] with bootstrapping techniques [69]. 

## 3. Results

### 3.1. Preliminary Analyses: Who Is Vaccine Hesitant and Who Uses Social Media as a Source of Information?

We found great variations in adherence to the four vaccinations (see Table 1). While 77.1% of respondents were favorable to vaccines in general, only 61.4% were favorable to HPV vaccination, whereas 84.6% were favorable to the measles vaccine. We also found differences in the type of hesitancy expressed by respondents (clear negative judgment vs. doubt).

### 3.2. Vaccine Typologies

As an additional tool to explore descriptive and inferential analyses involving VH, we collapsed this measure into three categories: “non-hesitant”, “hesitant but pro-vaccine”, and “distrustful of vaccination” categories. “Non-hesitant” individuals represented those favorable to vaccines in general and to all four vaccines listed (measles, hepatitis B, influenza, and HPV; 37.9% of sample; *n* = 3477). “Hesitant but pro-vaccine” respondents reflected those favorable of vaccines in general but unfavorable or uncertain about at least one of the four vaccines listed (38.8% of the sample; *n* = 3588). “Distrustful of vaccination” individuals include those respondents not favorable to vaccines in general or who were uncertain about them (22.9% of sample, *n* = 2102). Notably, only 6.8% of respondents were unfavorable or hesitant to all vaccines.

Table 2 provides a descriptive breakdown of social media information preference across different demographic and central study variables (institutional trust, vaccine hesitancy). Among all participants, 23.1% (*n* = 2120) noted social media as being one of three primary sources for information. Recourse to social media was strongest among those with lower income, those with less institutional trust, and younger participants. Consequently, these social groups were over-represented among the social media users: 61.2% were among the two lower quartiles for institutional trust, 45.6% earned 2000 euros a month or less (vs. 34.8%), and 48.4% were aged less than 35 (vs. 24.9% of the sample). 

Descriptive statistics provide a first assessment of the relationship between social media use and vaccine hesitancy. Social media users are more likely to be among those with a high vaccine hesitancy score (32.9% are in the highest quartile for vaccine hesitancy) and among the distrustful of vaccines group (34.7% are in this group). However, social media users only compose a minority within each of these groups (31.4% and 35.3% respectively). 

Notably, the two variables seem to have a combined effect. Within the first trust quartile, those who use social media seem to be more likely than those who do not to be among the most vaccine-hesitant quartile and among the distrustful of vaccination group (49.9% vs. 39.6% and 50.5% vs. 36.5%, respectively). Also, those who were most likely to be vaccine hesitant were likely to reflect both a combination of preference for information via social media and distrust in institutions. Indeed, among the people in the most vaccine hesitant quartile (Q4) who cite social media as one of their main sources of information, 77.8% were in the two quartiles most distrustful of institutions and only 49.9% were in the most trustful quartile. For the distrustful of vaccination who cite social media as one of their main sources of information, these proportions were 80.1% and 47.2%, respectively.

Overall, these descriptive findings provide frequency comparisons of vaccine hesitancy groupings across PISM and institutional trust categorizations. The subsequent analyses involve formal inferential tests of these relationships. 

### 3.3. Central Analyses

We first predicted that a significant negative relationship would exist between PISM and institutional trust. Because the social media measure is dichotomous, for interpretation purposes, we report the multiple regression results predicting institutional trust. After controlling for demographic variables, the regression analyses confirmed a significant, negative relationship between PISM and institutional trust (*B* = −0.45, *p* < 0.01, Δ*R*^2^ = 0.01). 

### 3.4. Predicting Vaccine Hesitancy

Preliminary correlation tests showed that institutional trust was negatively associated with vaccine hesitancy (*r* = 0.38, *p* < 0.01), while PISM positively correlated with vaccine hesitancy (*r* = 0.13, *p* < 0.01). Follow-up regression analyses show that both preference for information via social media (*B* = 0.10, *p* < 0.05) and institutional trust (*B* = −0.32, *p* < 0.01) significantly predicted VH (Δ*R*^2^ = 0.12, *p* < 0.01, see Table 3). PISM was an independent, negative predictor of VH, while institutional trust was an independent, positive predictor of VH. In terms of predictor power, the analyses indicate that social media was a relatively weak contributor to VH, while institutional trust had relatively stronger explanatory power. 

### 3.5. Mediation Analyses 

**Figure 1:**
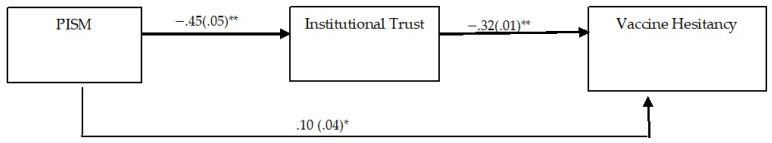
Institutional trust as mediator between preference for information via social media (PISM) and vaccine hesitancy. note: * = *p* < 0.05, ** = *p* < 0.01.

The second goal of this study involved testing competing mediating processes involving PISM, institutional trust, and VH. The first test revealed a significant indirect effect of PISM on VH through institutional trust (*B* = 0.14, *p* < 0.01; 95% confidence interval: 0.11 to 0.18; proportion mediated is 0.59). Reversing the sequential order of trust and social media use also revealed a significant indirect effect of institutional trust on VH through PISM (*B* = −0.002, *p* < 0.05; 95% confidence interval: −0.004 to 0.00; proportion mediated is 0.01). A comparison of these two mediating models highlights the greater support for institutional trust as a mediator between PISM and VH. Figure 1 provides a visual depiction of this relationship. 

### 3.6. Typology Assessments 

As noted above, we collapsed the VH measure into three categories. For clarity purposes, the following analyses examine how central study variables predict “non-hesitant” and “distrustful of vaccination” categorization. Results of the first multinomial logistic regression analysis showed that increased institutional trust predicted greater likelihood of being non-hesitant towards vaccination (*OR* = 1.41, *p* < 0.01), whereas PISM was not associated with likelihood of being non-hesitant (*OR* = 0.94 *p* > 0.05; model χ^2^ = 0.08; see Table 4). 

**Figure 2:**
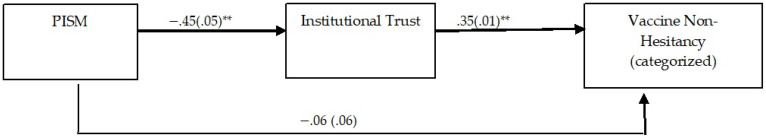
Institutional trust as mediator between preference for information via social media (PISM) and vaccine non-hesitancy (categorized). note: ** = *p* < 0.01.

Based on these findings, we ran one mediation test to assess whether social media use indirectly predicted likelihood of being non-hesitant via institutional trust. Findings revealed a significant indirect effect of PISM on likelihood of being non-hesitant through institutional trust (*OR* = −0.03, *p* < 0.01; 95% confidence interval: −0.04 to −0.03; proportion mediated is: 0.71; see Figure 2). 

Separate analyses explored how the central study variables predicted the likelihood of being distrustful of vaccination. Results of multinomial logistic regression analysis showed that increased institutional trust predicted a lower likelihood of being distrustful of vaccination (*OR* = 0.67, *p* < 0.01), whereas PISM predicted an increased likelihood of being distrustful of vaccination (*OR* = 1.33, *p* < 0.01; model χ^2^ = 0.14). In combination with the multinomial regression analyses reported above, we draw two conclusions. First, institutional trust operates to both increase the likelihood of vaccine non-hesitancy and reduce the likelihood of being in the distrustful of vaccination categorization. Second, and in contrast to institutional trust, PISM operates as an independent positive contributor to the distrustful of vaccination categorization, whereas it does not contribute to non-hesitancy categorization. Finally, although the results indicate these two measures predict VH categorization, the examination of odds ratios (OR) suggests that neither variable are strong contributors to VH typology. 

**Figure 3:**
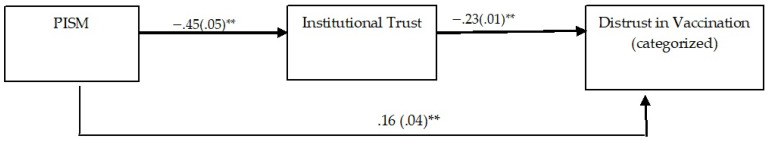
Institutional trust as a mediator between preference for information via social media (PISM) and distrust in vaccination (categorized). note: ** = *p* < 0.01.

To assess indirect effects of these measures on the distrustful of vaccination categorization, we explored two mediation paths. Results of the first test analysis revealed a significant indirect effect of PISM on the likelihood of being distrustful of vaccination through institutional trust (*OR* = 0.03, *p* < 0.01; 95% confidence interval: 0.02 to 0.03; proportion mediated is 0.39). The second indirect effect indicated a weaker yet significant mediation effect of institutional trust on the distrustful of vaccination categorization through PISM (*OR* = −0.001, *p* < 0.01; 95% confidence interval: −0.001 to 0.00; proportion mediated is 0.02). Overall, the mediation tests again indicate stronger support for institutional trust as the intervening variable in the relationship between social media information use and distrustful of vaccination categorization. Figure 3 provides a visual depiction of this relationship.

### 3.7. Post Hoc Analyses

We performed additional analyses to assess whether any interactions emerged between institutional trust and PISM in predicting VH. Results yielded no significant interaction effects. 

## 4. Discussion

### 4.1. Main Findings

This study examined how institutional trust and social media information preferences contribute to vaccine hesitancy in France. Across four waves among nationally representative survey populations, our findings indicate that lower levels of institutional trust act as a key contributor to increased VH. In addition, preference for information via social media (PISM) emerged as a significant, albeit weaker, positive predictor of VH. When examining mediation pathways, our results strongly support trust as a direct antecedent to VH, while PISM indirectly contributes to VH via trust. Typology classifications of “vaccine-hesitant” and “non-hesitant” groups offer additional insights on the role of these two predictors. Preliminary descriptive analyses show that respondents who display PISM only represent a minority among the vaccine hesitant and that most vaccine-hesitant respondents who display PISM also display lower than average trust in institutions. Inferential tests show that PISM had no direct relationship to “non-hesitant” classification; rather, this relationship was fully mediated through institutional trust. Conversely, PISM emerged as a strong, direct contributor to being part of the distrustful vaccination group. 

### 4.2. Implications 

Our study contributes to the current debate over how the circulation of false information on social media affects public attitudes to vaccines and science more generally. Our findings offer some support for arguments that the more direct relationships between social media exposure and VH may be overstated [70] and that too much emphasis might be put on social media use as an explanation for anti-science attitudes [50,71]. We found that few people turn primarily to social media for information, and that even among them, only a minority becomes particularly vaccine hesitant. This can be explained in several complementary ways. In France, as in many other countries, most people—even heavy social media users—are particularly distrustful of the information that circulates on social media [52]. Also, the circulation of information on social media tends to be segmented; meaning that different users will come across different types of information [72]. In the case of vaccination in France, a recent study of the spread of vaccine critical contents on Twitter during the COVID-19 epidemic found that vaccine critical activists had a limited ability to reach a wide audience, despite their intense activity [53]. A similar study conducted before the epidemic found similar results, as well as that defenders of vaccines tend to be retweeted by a greater number of users and that mainstream media occupy an important share of discussions [48]. Several recent studies on social media content suggest that the balance between defenders and critics of vaccines might have tipped in favor of the former [48,73,74]. Part of the explanation for the limited association between preference for social media and VH could therefore be a surge in pro-vaccination mobilization on social media and changes made by platforms to limit the spread of misinformation. 

Our findings—suggesting that preference for social media offers a limited explanation of vaccine hesitancy—are consistent with research documenting that France is among the most vaccine hesitant western nations while ranking low in social media use [75]. In the case of France, the prevalence of VH would mostly be explained by the combination of a structural distrust of public authorities and the recent emergence of public controversies surrounding vaccination which have made vaccines an object of this distrust [2].

However, the debate over the effects of the recent shift in public preferences for information from non-traditional media sources should not be reduced to the question of whether these effects are exaggerated versus understated. By focusing on those who have a preference for social media as a source of information, our study provides some elements to this debate, albeit in a limited manner. As several qualitative studies conducted in France have shown, even occasional social media use can lead to some parents encountering vaccine-critical information. This in turn triggers information-seeking processes that result in doubt or rejection of one or more vaccines [17,76]. Given how vaccine critics regularly appear in mainstream media—as has been the case in France in the past ten to twenty years [77]—people connect information on social media to information from other sources as well as interpret it against the backdrop of their worldviews and personal experience. Research on the role of social media in VH and public attitudes to science more generally is increasingly shifting attention to these questions pertaining to the way social media use acts as a cog in a set of complex mechanisms combining media practices, beliefs, worldviews, and structural factors [78,79]. Our study contributes to this movement by examining the complex inter-relations between institutional trust, preference for social media as a source of information, and vaccine hesitancy. In particular, one of the crucial findings highlights how these different phenomena operate in unison. The vaccine hesitant who prefer social media also distrust institutions. 

Our study also suggests pathways to advance understanding of the relationship between these complex phenomena, such as further investigation of generational differences in social media use [80]. Indeed, we found that the young are more vaccine hesitant and have a much higher PISM. While we did not find a strong generational gap when it comes to distrust in institutions in general, many studies focusing on trust in political actors, politicization, and participation to politics in France have found that people currently aged less than 30–40 are particularly wary of politicians and public institutions [65]. In France, the generation that grew up with the rise of social media also grew up with the economic crisis of 2008, rising inequalities, the rise of the Far Right and the Far Left, the demise of the traditional opposition between Center Right and Center Left parties, and the multiplication of vaccine-related controversies. Investigating the generational differences in social media practices and how they intersect with issues of trust, ideology, public participation, and health trajectories could help better understand exactly how social media use can affect public attitudes to vaccines and science more generally.

In France, vaccine-related controversies over the past 25 years have contributed to this distrust in vaccines. Consequently, institutional trust—impacted itself by faults in vaccine-related campaigns—drives VH. While France is not unique in these relationships, the fact that French respondents frequently report higher VH levels than other comparable nations [10,11,12] highlights the importance of clear, transparent messaging from French government/public health entities. 

The concern over how social media use impacts VH reflects a more recent shift in public preferences for information from non-traditional media sources. Our findings suggest that the association between social media and VH can at least partly be explained by how social media contributes to decreased institutional trust. Whereas traditional media sources are inclined to reference and/or support information provided by national institutions, social media may offer an outlet to those dissatisfied with these centralized sources. Consequently, social media may reinforce existing distrust and trigger new doubts in the actions of national institutions. 

### 4.3. Typologies 

By creating “distrustful of vaccination” and “non-hesitant” categories, we offer a distinct assessment of how institutional trust and PISM contribute to VH identifications. Whereas PISM modestly predicts “degree” of vaccine hesitancy, a clearer reflection of its role on vaccine-related attitudes emerges with the link between PISM and the likelihood of individuals “grouped” as distrustful of vaccination, representing those with concerns over vaccines in general. Individuals in this category do not discriminate across specific vaccines, but rather are likely to express hesitancy to any vaccine. In this regard, social media can potentially have a more generalizable impact on vaccine hesitancy beyond any specific vaccine concerns (e.g., HPV, measles). Conversely, levels of institutional trust increase the likelihood of non-hesitancy categorization (no vaccine-related concerns overall or to any specific vaccine) as well as decreasing distrustful of vaccination designation. 

Arguably, the most pronounced representation of the interrelationship between PISM and institutional trust on VH emerges when examining these typologies. PISM alone does not contribute to non-vaccine hesitant categorization, but rather, its impact is fully indirect through institutional trust. Thus, while PISM does not directly reduce non-vaccine categorization, it indirectly operates to decrease this designation by reducing trust. When investigating those who are non-hesitant, researchers should explore how often, and in what selective ways, these individuals utilize information provided by social media sources. Consistent with prior research on the limited spread of social media misinformation [5], these individuals may be more inclined to seek out, consume, and ultimately believe legitimate/credible vaccine-related information. In terms of “vaccine hesitancy” grouping, PISM holds up as a direct factor increasing VH, while also having indirect effects via trust. Among these individuals, identifying the most problematic social media that acts to reinforce blanket concerns over vaccination is central to ongoing campaign efforts.

### 4.4. Limitations

Our analysis presents the usual limitations of survey-based studies. The literature on fake news and mis-/dis-information has underlined the gap between respondents’ declarations regarding social media use and the time they actually spend on social media [50,51]. To reflect this, we focused on preference for social media information rather than declaration regarding the frequency of social media consultation. The timing of the study can also bear heavily on responses to survey-based questionnaires. Social media use and preferences for sources of information are in constant evolution, especially in France [52]. This study sheds light on the relationship between preference for social media as sources of information, institutional trust, and vaccine hesitancy at a particular, but crucial moment in time both for vaccines and for social media: the second year of the COVID-19 epidemic. Similar analyses on data pertaining to the period pre-COVID-19 and to future periods are necessary to build a more fine-grained and comprehensive understanding of the dynamic relationship between these factors. Vaccine hesitancy is context dependent, and previous pandemics have seen dramatic shifts in attitudes to vaccines in general [81]. Notably, COVID-19 does not seem to have dramatically changed attitudes to vaccines. In our study, the share of respondents favorable to vaccines in general is modestly higher than studies conducted pre-pandemic [82]. In sum, our results indicate that (a) a majority of French people can be defined as hesitant, (b) a minority reject all vaccines, and (c) most hesitant people do not distrust vaccines in general. These findings are consistent with studies conducted before and during the COVID-19 epidemic [2,20].

## 5. Conclusions

Vaccine hesitancy (VH) continues to pose significant public health challenges in French society. This project explored how institutional trust and preference for information via social media (PISM) drive hesitancy. Across a large, nationally represented population, our findings show that PISM and trust are strongly correlated measures, with both independently predicting VH. Subsequent mediation tests show that social media operates as primarily an indirect impact on VH through trust. Additional tests involving VH and non-VH typologies revealed that institutional trust consistently predicts greater general support for vaccines and reduced distrust in vaccination. Conversely, PISM directly drives vaccine distrust, with its impact on non-hesitancy fully mediated by institutional trust. Overall, these findings point to the relevance for researchers and public health deciders to address the nature by which people utilize social media information resources and how that interacts with levels of trust for national institutions. 

## Figures and Tables

**Table 1:** Participant response to vaccine hesitancy items.

	Very Favorable or Somewhat Favorable	Don’t Know	Very Unfavorable or Somewhat Unfavorable
Vaccines in general	77.1%	5.0%	17.9%
HPV	61.4%	24.4%	14.2%
Flu	63.3%	8.1%	28.6%
Hepatitis B	69.0%	13.6%	17.4%
Measles	84.6%	8.6%	6.8%

**Table 2:** Frequency of preference for information via social media (PISM) by category.

		% Sample	% Who Put Social Media as One of Their Three Main Sources of Information (Row Percentage)	% Among Those Who Put Social Media as One of Their Three Main Sources of Information (Column Percentage)
Sex	Female	52.4%	26.0%	58.7%
	Male	47.6%	20.1%	41.3%
Age	18–34	24.9%	45.0%	48.4%
	35–64	48.5%	20.9%	43.7%
	65 and older	26.6%	6.9%	8.0%
Education	Less than HS	33.0%	25.9%	36.8%
	HS degree	27.7%	23.9%	28.5%
	Bachelor’s degree	27.7%	20.9%	25.0%
	Master’s degree	11.6%	19.3%	9.7%
Income	1—0–1000 euros	8.3%	36.5%	13.0%
	2—1000–2000 euros	26.5%	28.6%	32.6%
	3—2000–4000 euros	40.4%	20.0%	34.8%
	4—4000 euros and more	12.7%	13.3%	7.3%
	5—NA	12.2%	23.4%	12.3%
score_confiance_quartiles	No trust Q1	25.0%	30.2%	32.5%
	Low trust Q2	25.1%	26.5%	28.7%
	High trust Q3	24.9%	20.3%	21.8%
	Strong trust Q4	25.1%	15.8%	17.1%
Vaccine hesitancy score	Q1	25.6%	17.4%	19.2%
	Q2	24.8%	18.4%	19.7%
	Q3	25.3%	25.9%	28.2%
	Q4	24.3%	31.4%	32.9%
Typology	Non vaccine hesitancy	38.1%	19.0%	31.2%
	Pro vaccine but selective	38.6%	20.2%	33.7%
	Distrustful of vaccines	22.9%	35.3%	34.7%

**Table 3:** Summary of hierarchical regression analyses for variables predicting vaccine hesitancy (VH).

	*B*	SE	*R* ^2^	∆*R*^2^
Step1: Control measures			0.07 **	
Sex (Male)	−0.35 **	0.03		
Age				
35–49	−0.14 **	0.05		
50–64	−0.28 **	0.05		
65+	−0.75 **	0.05		
Education				
HS degree	−0.18 **	0.04		
Bachelor’s degree	−0.27 **	0.05		
Master’s degree	−0.50 **	0.06		
Income				
EUR 0–2000	0.15 *	0.06		
EUR 2000–4000	−0.07	0.06		
>EUR 4000	−0.37 **	0.07		
Step 2: Main study variables				0.12 **
Institutional trust	0.10 *	0.04		
PISM	−0.32 **	0.01		

Note: Regression coefficients are unstandardized. PISM = preference for information via social media; * = *p* < 0.05, ** = *p* < 0.01.

**Table 4:** Summary of hierarchical logistic regression analyses for variables predicting vaccine hesitancy and vaccine non-hesitancy categorizations.

	Vaccine Hesitancy		Vaccine Non-Hesitancy	
	OR	95% CI	OR	95% CI
Demographics/controls				
Sex (male)	0.81 **	0.73–0.90	1.31 **	1.20–1.43
Age				
35–49	0.69 **	0.61–0.79	1.19	1.05–1.35
50–64	0.48 **	0.42–0.55	1.26	1.11–1.43
65+	0.27 **	0.23–0.32	1.56 **	1.38–1.76
Education				
HS degree	0.63 **	0.56–0.72	1.34 **	1.19–1.50
Bachelor’s degree	0.52 **	0.46–0.59	1.61 **	1.44–1.79
Master’s degree	0.25 **	0.16–0.39	1.74 **	1.33–2.28
Income				
EUR 0–2000	1.03	0.88–1.20	1.13	.97–1.31
EUR 2000–4000	0.67 **	0.56–0.78	1.28 **	1.11–1.48
>EUR 4000	0.44 **	0.35–0.56	1.78 **	1.49–2.12
Central predictors				
Institutional trust	0.10 *	0.04	1.41 **	1.38–1.45
PISM	−0.32 **	0.01	0.94	0.84–1.06

Note: OR = odds ratio. An odds ratio greater than one indicates respondents were more likely to be categorized as vaccine hesitant/non-hesitant. An odds ratio of less than one indicates that respondents were less likely to be categorized as vaccine hesitant/non-hesitant. PISM = preference for information via social media; * = *p* < 0.05, ** = *p* ≤ 0.01.

## Data Availability

The descriptive data/analyses for this study are available at https://doi.org/10.6084/m9.figshare.23681031. All other data is available upon request.

## References

[1] Ward J.K., Colgrove J., Verger P. (2018). Why France is making eight new vaccines mandatory. Vaccine.

[2] Ward J.K., Peretti-Watel P. (2020). Understanding vaccine mistrust: From perception bias to controversies. Rev. Fr. De Sociol..

[3] Dunn A.G., Surian D., Leask J., Dey A., Mandl K.D., Coiera E. (2017). Mapping information exposure on social media to explain differences in HPV vaccine coverage in the United States. Vaccine.

[4] Featherstone J.D., Zhang J. (2020). Feeling angry: The effects of vaccine misinformation and refutational messages on negative emotions and vaccination attitude. J. Health Commun..

[5] Guess A.M., Lockett D., Lyons B., Montgomery J.M., Nyhan B., Reifler J. (2020). “Fake news” may have limited effects on political participation beyond increasing beliefs in false claims. Harv. Kennedy Sch. Misinf. Rev..

[6] Dubé È., Ward J.K., Verger P., MacDonald N.E. (2021). Vaccine Hesitancy, Acceptance, and Anti-Vaccination: Trends and Future Prospects for Public Health. Annu. Rev. Public Health.

[7] Bedford H., Attwell K., Danchin M., Marshall H., Corben P., Leask J. (2018). Vaccine hesitancy, refusal and access barriers: The need for clarity in terminology. Vaccine.

[8] Peretti-Watel P., Larson H.J., Ward J.K., Schulz W.S., Verger P. (2015). Vaccine Hesitancy: Clarifying a Theoretical Framework for an Ambiguous Notion. PLoS Curr..

[9] MacDonald N.E., The SAGE Working Group on Vaccine Hesitancy (2015). Vaccine hesitancy: Definition, scope and determinants. Vaccine.

[10] Gallop World Poll Welcome Global Monitor 2018. https://wellcome.org/reports/wellcome-global-monitor/2018.

[11] Larson H.J., De Figueiredo A., Xiahong Z., Schulz W.S., Verger P., Johnston I.G., Cook A.R., Jones N.S. (2016). The State of Vaccine Confidence 2016: Global Insights Through a 67-Country Survey. EBioMedicine.

[12] Rabesandratana T. (2019). France Most Skeptical about Science and Vaccines, Global Survey Finds. https://www.science.org/content/article/france-most-skeptical-about-science-and-vaccines-global-survey-finds.

[13] Ward J.K., Peretti-Watel P., Bocquier A., Seror V., Verger P. (2019). Vaccine hesitancy and coercion: All eyes on France. Nat. Immunol..

[14] Ipsos (2020). Global Attitudes on a COVID-19 Vaccine: Ipso Survey for the World Economic Forum. https://www.ipsos.com/en/global-attitudes-COVID-19-vaccine-december-2020.

[15] Sante Publique France (2022). InfoCovidFrance. https://www.santepubliquefrance.fr/dossiers/coronavirus-covid-19/english-coronavirus-key-numbers-for-covid-19-and-its-evolution-in-france-and-across-the-world.

[16] (2022). COVIREIVAC. http://www.orspaca.org/recherche/enqu%C3%AAte-en-population-g%C3%A9n%C3%A9rale-sur-lacceptabilit%C3%A9-dun-vaccin-contre-la-covid-19.

[17] Peretti-Watel P., Ward J.K., Vergelys C., Bocquier A., Raude J., Verger P. (2019). *‘I Think I Made the Right Decision … I Hope I’m Not Wrong’*. Vaccine hesitancy, commitment and trust among parents of young children. Sociol. Health Illn..

[18] Bocquier A., Fressard L., Cortaredona S., Zaytseva A., Ward J., Gautier A., Peretti-Watel P., Verger P. (2018). Social differentiation of vaccine hesitancy among French parents and the mediating role of trust and commitment to health: A nationwide cross-sectional study. Vaccine.

[19] Verger P., Peretti-Watel P., Gagneux-Brunon A., Botelho-Nevers E., Sanchez A., Gauna F., Fressard L., Bonneton M., Launay O., Ward J.K. (2021). Acceptance of childhood and adolescent vaccination against COVID-19 in France: A national cross-sectional study in May 2021. Hum. Vaccines Immunother..

[20] Bajos N., Spire A., Silberzan L., Sireyjol A., Jusot F., Meyer L., Franck J.-E., Warszawski J. (2022). The EpiCov study group When Lack of Trust in the Government and in Scientists Reinforces Social Inequalities in Vaccination Against COVID-19. Front. Public Health.

[21] Bridgman A., Merkley E., Loewen P.J., Owen T., Ruths D., Teichmann L., Zhilin O. (2020). The causes and consequences of COVID-19 misperceptions: Understanding the role of news and social media. Harv. Kennedy Sch. Misinf. Rev..

[22] Betsch C., Brewer N.T., Brocard P., Davies P., Gaissmaier W., Haase N., Leask J., Renkewitz F., Renner B., Reyna V.F. (2012). Opportunities and challenges of Web 2.0 for vaccination decisions. Vaccine.

[23] Gauna F., Verger P., Fressard L., Jardin M., Ward J.K., Peretti-Watel P. (2023). Vaccine hesitancy about the HPV vaccine among French young women and their parents: A telephone survey. BMC Public Health.

[24] (2023). Service-Public.fr. Le Site Officiel de L’administration Francaise. https://www.service-public.fr/particuliers/actualites/A15259?lang=en.

[25] Newton K., Stolle D., Zmerli S., Uslaner E.M. (2018). Social and political trust. The Oxford Handbook of Social and Political Trust.

[26] Uslaner E.M. (2002). The Moral Foundations of Trust.

[27] Rowe R., Calnan M. (2006). Trust relations in health care—The new agenda. Eur. J. Public Health.

[28] Van Bavel J.J., Baicker K., Boggio P.S., Capraro V., Cichocka A., Cikara M., Crockett M.J., Crum A.J., Douglas K.M., Druckman J.N. (2020). Using Social and Behavioural Science to Support COVID-19 Pandemic Response. Nat. Hum. Behav..

[29] Blair R.A., Morse B.S., Tsai L.L. (2017). Public health and public trust: Survey evidence from the Ebola Virus Disease epidemic in Liberia. Soc. Sci. Med..

[30] Rubin G.J., Amlôt R., Page L., Wessely S. (2009). Public perceptions, anxiety, and behaviour change in relation to the swine flu outbreak: Cross sectional telephone survey. BMJ.

[31] Vinck P., Pham P.N., Bindu K.K., Bedford J., Nilles E.J. (2019). Institutional trust and misinformation in the response to the 2018–19 Ebola outbreak in North Kivu, DR Congo: A population-based survey. Lancet Infect. Dis..

[32] Karafillakis E., Simas C., Jarrett C., Verger P., Peretti-Watel P., Dib F., De Angelis S., Takacs J., Ali K.A., Pastore Celentano L. (2019). HPV vaccination in a context of public mistrust and uncertainty: A systematic literature review of determinants of HPV vaccine hesitancy in Europe. Hum. Vaccines Immunother..

[33] Ward J.K. (2016). Rethinking the antivaccine movement concept: A case study of public criticism of the swine flu vaccine’s safety in France. Soc. Sci. Med..

[34] Wilson R.J.I., Vergélys C., Ward J., Peretti-Watel P., Verger P. (2020). Vaccine hesitancy among general practitioners in Southern France and their reluctant trust in the health authorities. Int. J. Qual. Stud. Health Well-Being.

[35] Schwarzinger M., Verger P., Guerville M.-A., Aubry C., Rolland S., Obadia Y., Moatti J.-P. (2010). Positive attitudes of French general practitioners towards A/H1N1 influenza-pandemic vaccination: A missed opportunity to increase vaccination uptakes in the general public?. Vaccine.

[36] Verger P., Fressard L., Collange F., Gautier A., Jestin C., Launay O., Raude J., Pulcini C., Peretti-Watel P. (2015). Vaccine Hesitancy Among General Practitioners and Its Determinants During Controversies: A National Cross-sectional Survey in France. EBioMedicine.

[37] Buchanan R., Beckett R.D. (2014). Assessment of vaccination-related information for consumers available on Facebook^®^. Health Inf. Libr. J..

[38] Smith T.C. (2017). Vaccine Rejection and Hesitancy: A Review and Call to Action. Open Forum Infect. Dis..

[39] Bäckström C., Larsson T., Wahlgren E., Golsäter M., Mårtensson L.B., Thorstensson S. (2017). ‘It makes you feel like you are not alone’: Expectant first-time mothers’ experiences of social support within the social network, when preparing for childbirth and parenting. Sex. Reprod. Healthc..

[40] Center for Countering Digital Hate (2020). Failure to Act: How Tech Giants Continue to Defy Calls to Rein in Vaccine Misinformation.

[41] Ache K.A., Wallace L.S. (2008). Human Papillomavirus Vaccination Coverage on YouTube. Am. J. Prev. Med..

[42] Briones R., Nan X., Madden K., Waks L. (2012). When Vaccines Go Viral: An Analysis of HPV Vaccine Coverage on YouTube. Health Commun..

[43] Bradshaw A.S., Shelton S.S., Wollney E., Treise D., Auguste K. (2020). Pro-Vaxxers Get Out: Anti-Vaccination Advocates Influence Undecided First-Time, Pregnant, and New Mothers on Facebook. Health Commun..

[44] Jamison A.M., Qi S., AlKulaib L., Chen T., Benton A., Quinn S.C., Dredze M. (2018). Weaponized Health Communication: Twitter Bots and Russian Trolls Amplify the Vaccine Debate. Am. J. Public Health.

[45] Lazer D.M.J., Baum M.A., Benkler Y., Berinsky A.J., Greenhill K.M., Menczer F., Metzger M.J., Nyhan B., Pennycook G., Rothschild D. (2018). The science of fake news. Science.

[46] Shi W., Liu D., Yang J., Zhang J., Wen S., Su J. (2020). Social Bots’ Sentiment Engagement in Health Emergencies: A Topic-Based Analysis of the COVID-19 Pandemic Discussions on Twitter. Int. J. Environ. Res. Public Health.

[47] Varol O., Ferrara E., Davis C., Menczer F., Flammini A. Online human-bot interactions: Detection, estimation, and characterization. Proceedings of the International AAAI Conference on Web and Social Media.

[48] Gargiulo F., Cafiero F., Guille-Escuret P., Seror V., Ward J.K. (2020). Asymmetric participation of defenders and critics of vaccines to debates on French-speaking Twitter. Sci. Rep..

[49] Martin S., Kilich E., Dada S., Kummervold P.E., Denny C., Paterson P., Larson H.J. (2020). “Vaccines for pregnant women…?! Absurd”—Mapping maternal vaccination discourse and stance on social media over six months. Vaccine.

[50] Altay S., Nielsen R.K., Fletcher R. (2022). Quantifying the “infodemic”: People turned to trustworthy news outlets during the 2020 coronavirus pandemic. J. Quant. Descr..

[51] Cordonier L., Brest A. Comment Les Français s’ Informent-Ils sur Internet? Analyse des Comportements D’information et de Désinformation en Ligne. Fondation Descartes. https://www.fondationdescartes.org/wp-content/uploads/2021/03/Etude_Information_Internet_FondationDescartes_2021.pdf.

[52] Kantar Public One Point (2022). La Confiance des Français dans les Media: Résultats de L’édition 2022 du Baromètre La Croix/Kantar Public Onepoint. https://www.kantarpublic.com/fr/barometres/barometre-de-la-confiance-des-francais-dans-les-media/barometre-2022-de-la-confiance-des-francais-dans-les-media.

[53] Faccin M., Gargiulo F., Atlani-Duault L., Ward J.K. (2022). Assessing the influence of French vaccine critics during the two first years of the COVID-19 pandemic. PLoS ONE.

[54] Dredze M., Broniatowski D.A., Hilyard K.M. (2016). Zika vaccine misconceptions: A social media analysis. Vaccine.

[55] Bandura A. (1986). Social Foundations of Thought and Action: A Social Cognitive Theory.

[56] Zimand-Sheiner D., Kol O., Frydman S., Levy S. (2021). To Be (Vaccinated) or Not to Be: The Effect of Media Exposure, Institutional Trust, and Incentives on Attitudes toward COVID-19 Vaccination. Int. J. Environ. Res. Public Health.

[57] Mari S., Gil de Zúñiga H., Suerdem A., Hanke K., Brown G., Vilar R., Boer D., Bilewicz M. (2021). Conspiracy Theories and Institutional Trust: Examining the Role of Uncertainty Avoidance and Active Social Media Use. Political Psychol..

[58] Tsfati Y., Tukachinsky R., Peri Y. (2009). Exposure to News, Political Comedy, and Entertainment Talk Shows: Concern about Security and Political Mistrust. Int. J. Public Opin. Res..

[59] Walter N., Brooks J.J., Saucier C.J., Suresh S. (2020). Evaluating the Impact of Attempts to Correct Health Misinformation on Social Media: A Meta-Analysis. Health Commun..

[60] Wang Y., McKee M., Torbica A., Stuckler D. (2019). Systematic Literature Review on the Spread of Health-related Misinformation on Social Media. Soc. Sci. Med..

[61] Jennings W., Stoker G., Bunting H., Valgarðsson V.O., Gaskell J., Devine D., McKay L., Mills M.C. (2021). Lack of Trust, Conspiracy Beliefs, and Social Media Use Predict COVID-19 Vaccine Hesitancy. Vaccines.

[62] Ball-Rokeach S.J. (1985). The origins of individual media-system dependency. Commun. Res..

[63] Miller J.M. (2020). Psychological, Political, and Situational Factors Combine to Boost COVID-19 Conspiracy Theory Beliefs. Can. J. Political Sci..

[64] Huurne E.T., Gutteling J. (2008). Information needs and risk perception as predictors of risk information seeking. J. Risk Res..

[65] Lardeux L., Tiberj V. (2021). Générations Désenchantées? Jeunes et Démocratie.

[66] Spire A. (2018). Résistances à L’impôt, Attachement à l’Etat-Enquête Sur les Contribuables Français.

[67] McKinley C.J., Lauby F. (2021). Anti-Vaccine Beliefs and COVID-19 Information Seeking on Social Media: Examining Processes Influencing COVID-19 Beliefs and Preventative Actions. Int. J. Commun-Us..

[68] Sobel M.E. (1982). Asymptotic Confidence Intervals for Indirect Effects in Structural Equation Models. Sociol. Methodol..

[69] Preacher K.J., Hayes A.F. (2004). SPSS and SAS procedures for estimating indirect effects in simple mediation models. Behav. Res. Methods Instrum. Comput..

[70] Edelstein M., Müller M., Ladhani S., Yarwood J., Salathé M., Ramsay M. (2020). Keep calm and carry on vaccinating: Is anti-vaccination sentiment contributing to declining vaccine coverage in England?. Vaccine.

[71] Scheufele D.A., Krause N.M., Freiling I. (2021). Misinformed about the “infodemic?” Science’s ongoing struggle with misinformation. J. Appl. Res. Mem. Cogn..

[72] Ross Arguedas A., Robertson C., Fletcher R., Nielsen R. (2022). Echo Chambers, Filter Bubbles, and Polarisation: A Literature Review.

[73] Johnson N.F., Velásquez N., Restrepo N.J., Leahy R., Gabriel N., El Oud S., Zheng M., Manrique P., Wuchty S., Lupu Y. (2020). The online competition between pro- and anti-vaccination views. Nature.

[74] Schmidt A.L., Zollo F., Scala A., Betsch C., Quattrociocchi W. (2018). Polarization of the vaccination debate on Facebook. Vaccine.

[75] (2022). Digimind Les Chiffres Essentiels Pour Comprendre les Réseaux Sociaux. https://landing.digimind.com/fr/guide-2021-chiffres-essentiels-social#form.

[76] Ward J.K., Crépin L., Bauquier C., Vergelys C., Bocquier A., Verger P., Peretti-Watel P. (2017). ‘I don’t know if I’m making the right decision’: French mothers and HPV vaccination in a context of controversy. Health Risk Soc..

[77] Cafiero F., Guille-Escuret P., Ward J.K. (2021). “I’m not an antivaxxer, but…”: Spurious and authentic diversity among vaccine critical activists. Soc. Netw..

[78] Krause N.M., Scheufele D.A., Freiling I., Brossard D. (2021). The Trust Fallacy: Scientists’ search for public pathologies is unhealthy, unhelpful, and ultimately unscientific. Am. Sci..

[79] Scheufele D., Krause N. (2019). Science audiences, misinformation, and fake news. Proc. Natl. Acad. Sci. USA.

[80] Guess A., Nagler J., Tucker J. (2019). Less than you think: Prevalence and predictors of fake news dissemination on Facebook. Sci. Adv..

[81] Peretti-Watel P., Raude J., Sagaon-Teyssier L., Constant A., Verger P., Beck F. (2014). Attitudes toward vaccination and the H1N1 vaccine: Poor people’s unfounded fears or legitimate concerns of the elite?. Soc. Sci. Med..

[82] Gautier A., Chemlal K., Jestin C. (2017). Adhésion à la vaccination en France: Résultats du baromètre santé 2016. Bull Epidémiol Hebd.

